# Biologically active, high levels of interleukin-22 inhibit hepatic gluconeogenesis but do not affect obesity and its metabolic consequences

**DOI:** 10.1186/s13578-015-0015-0

**Published:** 2015-05-30

**Authors:** Ogyi Park, Sung Hwan Ki, Mingjiang Xu, Hua Wang, Dechun Feng, Joseph Tam, Douglas Osei-Hyiaman, George Kunos, Bin Gao

**Affiliations:** Laboratory of Liver Diseases, NIAAA/NIH, 5625 Fishers Lane, Bethesda, MD 20892 USA; Laboratory of Physiologic Studies, National Institute on Alcohol Abuse and Alcoholism, National Institutes of Health, Bethesda, MD 20892 USA; Laboratory of Toxicology, College of Pharmacy, Chosun University, Gwangju, South Korea; Obesity and Metabolism Laboratory, The Institute for Drug Research, School of Pharmacy, Faculty of Medicine, The Hebrew University of Jerusalem, Jerusalem, 91120 Israel

**Keywords:** Obesity, Insulin resistance, Hyperglycemia, Cytokine, Liver

## Abstract

**Background:**

Interleukin-22 (IL-22), a cytokine with important functions in anti-microbial defense and tissue repair, has been recently suggested to have beneficial effects in obesity and metabolic syndrome in some but not in other studies. Here, we re-examined the effects of IL-22 on obesity, insulin resistance, and hepatic glucose metabolism.

**Results:**

Genetic deletion of IL-22 did not affect high-fat-diet (HFD)-induced obesity and insulin resistance. IL-22 transgenic mice with relatively high levels of circulating IL-22 (~600 pg/ml) were completely resistant to Concanavalin A-induced liver injury but developed the same degree of high fat diet (HFD)-induced obesity, insulin resistance, and fatty liver as the wild-type littermate controls. Similarly, chronic treatment with recombinant mouse IL-22 (rmIL-22) protein did not affect HFD-induced obesity and the associated metabolic syndrome. *In vivo* treatment with a single dose of rmIL-22 downregulated the hepatic expression of gluconeogenic genes and subsequently inhibited hepatic gluconeogenesis and reduced blood glucose levels both in HFD-fed and streptozotocin (STZ)-treated mice without affecting insulin production. *In vitro* exposure of mouse primary hepatocytes to IL-22 suppressed glucose production and the expression of gluconeogenic genes. These inhibitory effects were partially reversed by blocking STAT3 or the AMPK signaling pathway.

**Conclusion:**

Biologically active, high levels of IL-22 do not affect obesity and the associated metabolic syndrome. Acute treatment with IL-22 inhibits hepatic gluconeogenesis, which is mediated via the activation of STAT3 and AMPK in hepatocytes.

Interleukin-22 (IL-22) is probably the only cytokine that is produced by immune cells but does not directly target immune cells due to lack of IL-22 receptor 1 (IL-22R1) expression on these cells [1–6]. Instead, IL-22 mainly targets epithelial cells which express high levels of IL-22R1 [1–6]. Several types of immune cells have been reported to produce IL-22. These include Th17 cells, Th22 cells, activated NK and NKT cells and others [1–6]. The action of IL-22 is mediated via its binding to IL-10R2 and IL-22R1, followed by the activation of signal transducer and activator of transcription 3 (STAT3) and, to a lesser extent, activation of additional signaling pathways such as STAT1, STAT5, AKT, ERK, etc. [1–6]. IL-10R2 is ubiquitously expressed while IL-22R1 is expressed exclusively in epithelial cells (eg. hepatocytes), hepatic stellate cells [HSCs], and fibroblasts [1–7]. Accumulating evidence suggests that IL-22 plays a critical role in anti-microbial defense and tissue repair in various organs [1–6]. In the liver, through its action on hepatocytes, IL-22 has been shown to act as a hepatoprotective factor that protects against liver injury, fibrosis and steatosis via the activation of STAT3 in a variety of rodent models and patients [7–13]. IL-22 does not initiate liver cancer development but can promote proliferation of existing liver tumor cells via the activation of STAT3 [14–16]. Although it does not target immune cells, IL-22 may indirectly promote liver inflammation in diseased liver, such as in chronic viral hepatitis [17].

Numerous recent studies suggest that IL-22 modulates obesity and its metabolic consequences, but the results are inconsistent [18–24]. In an early study, injection of mice with adenovirus expressing IL-22, which produced super high levels of circulating IL-22, induced marked body weight loss and thymic atrophy in lean mice [18]. We also observed that IL-22 transgenic mice expressing super high levels of IL-22 (4000–7000 pg/ml) had much lower lean body weight [14]. It should be noted that serum IL-22 levels in healthy individuals and patients with various types of diseases rarely exceed 200 pg/ml [15, 25, 26]. Recently, Wang et al. [19] reported that treatment with high doses of long half-life IL-22Fc protein (50–100 μg/mouse, half-life t_1/2_ = 3.02 days, twice a week for 4 weeks) reduced body weight and ameliorated hyperglycemia and insulin resistance in obese, leptin receptor-deficient mice and mice fed a high-fat diet (HFD). Interestingly, Hasnain et al. [20] reported that chronic treatment with low doses of short half-life recombinant mouse IL-22 protein (rmIL-22)(20 ng/g or 100 ng/g, twice a week for 4 weeks) reduced body weight and alleviated metabolic complications caused by HFD in mice. However, Yang et al. [21] found that chronic treatment with rmIL-22 (300 ng/g, daily for 36 days) ameliorated fatty liver but did not affect body weight and metabolic parameters in HFD-fed mice. In contrast, Upadhyay et al. [22] observed that overexpression of IL-22 via hydrodynamic injection restored normal body weight and adiposity in lymphotoxin β receptor knockout mice. In addition, several studies suggested that T cell-derived IL-22 enhances IL-1β-mediated inflammation in human adipose tissue and reduces insulin sensitivity in human hepatocytes, promoting obesity and diabetes [23, 27].

In the current study, we analyzed the effects of endogenous IL-22 and chronic treatment with rmIL-22 as well as genetic overexpression of IL-22 on HFD-induced obesity and metabolic syndrome. Our results indicate that high circulating levels of transgenically expressed IL-22, chronic treatment with rmIL-22, or deficiency in endogenous IL-22 do not affect HFD-induced obesity and its metabolic consequences in mice. The effect of IL-22 on glucose metabolism in hepatocytes were also examined.

## Results

### Endogenous IL-22 does not play a role in regulating obesity, insulin resistance, and fatty liver disease induced by 10 weeks of HFD feeding

**Fig. 1:**
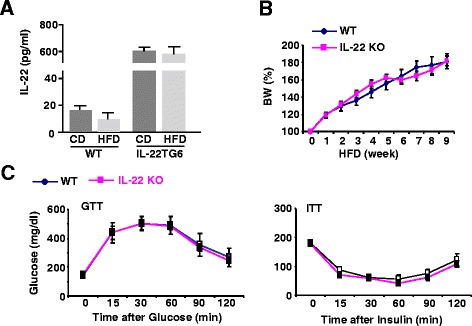
Endogenous IL-22 does not play a role in the development of obesity and insulin resistance induced by a HFD. **a** Two-month old IL-22TG6 mice and their littermate controls were fed a HFD or CD for 10 weeks. Serum IL-22 levels were measured. **b**, **c** Two-month old IL-22KO mice and their littermate controls were fed a HFD or CD for 10 weeks. Body weight was counted weekly (panel **b**); GTT and ITT were examined after 10 weeks feeding (panel **c**). CD; control diet; HFD: high-fat diet. Values represent the mean ± SEM (n = 6-12)

It was reported that basal serum levels of IL-22 were approximately 20 pg/ml in control diet-fed mice, and were decreased to 5 pg/ml in HFD-fed mice [28]. However, a recent study reported that serum levels of IL-22 were markedly increased after HFD diet feeding to approximately 1000 pg/ml compared to approximately 100 pg/ml in chow-fed mice (Extended data Fig. 1 in reference [19]). Here we found that serum IL-22 levels were relatively low (<20 pg/ml) in control- or HFD-fed mice with lower levels in HFD-fed mice than those in control-fed mice, while high levels of serum IL-22 were detected in IL-22TG6 mice (~600 pg/ml) (Fig. 1a). HFD feeding did not affect serum IL-22 levels in IL-22TG6 mice.

To determine the role of IL-22 in obesity and insulin resistance, we bred IL-22^+/−^ heterozygous mice to generate wild-type littermates and IL-22KO mice. These mice were fed a HFD or control diet for 10 weeks. As illustrated in Fig. 1b, both WT and IL-22KO mice had similar body weight gain, glucose intolerance, and insulin resistance after 10 weeks of HFD feeding. In addition, hepatic steatosis as determined by H&E staining and measurement of hepatic triglyceride levels was comparable between HFD-fed WT and IL-22KO mice (data not shown). This suggests that very low levels of endogenous IL-22 do not contribute to the pathogenesis of obesity, insulin resistance, and fatty liver disease in the 10-week HFD feeding model.

### Liver-specific IL-22TG6 mice with relatively high levels of circulating IL-22 (~600 pg/ml) are resistant to concanavalin A-induced liver injury but develop the same degree of HFD-induced obesity, insulin resistance, and fatty liver as wild-type littermates

**Fig. 2:**
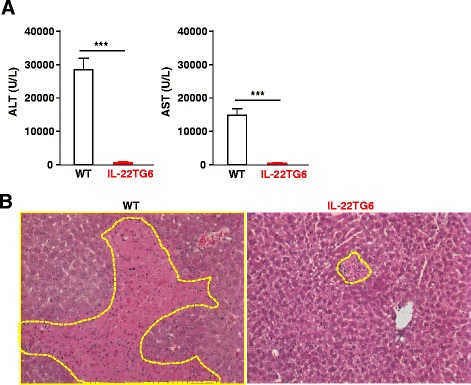
IL-22TG6 mice with high levels of circulating IL-22 (~600 pm/ml) are resistant to Con A-induced liver injury. WT and IL-22TG mice were injected with Con A (15 μg/g) for 24 h. **a** Serum ALT and AST levels were measured. **b** Representative H&E staining of liver tissues from mice treated with Con A for 24 h. Necrotic areas are indicated by dot circles. Values represent the mean ± SEM (n = 10-14). ***P < 0.001

The role of IL-22 in regulating obesity and metabolic syndrome was further examined in IL-22 transgenic mice, in which IL-22 expression is controlled by albumin promoter and enhancer [14, 29]. We obtained four lines of IL-22TG mice, including three lines (IL-22TG8, TG9, and TG15) that have super high levels of circulating IL-22 (4000–7000 pg/ml) and one line (IL-22TG6) that has relatively high levels of circulating IL-22 (~600 pg/ml) (Fig. 1a) [14, 29]. We have previously demonstrated that the three lines with super high levels of IL-22 were completely resistant to Con A-induced liver injury [14]. Here we report that IL-22TG6 mice were also completely resistant to Concanavalin A-induced liver injury, as demonstrated by the marked elevation of serum ALT and AST in WT mice but not in IL-22TG mice (Fig. 2a). Liver histology analysis revealed that WT mice had massive necrosis while IL-22TG6 mice only had a few of small necrotic areas (Fig. 2b). This suggests that high levels of IL-22 in IL-22TG6 are biologically active.

**Fig. 3:**
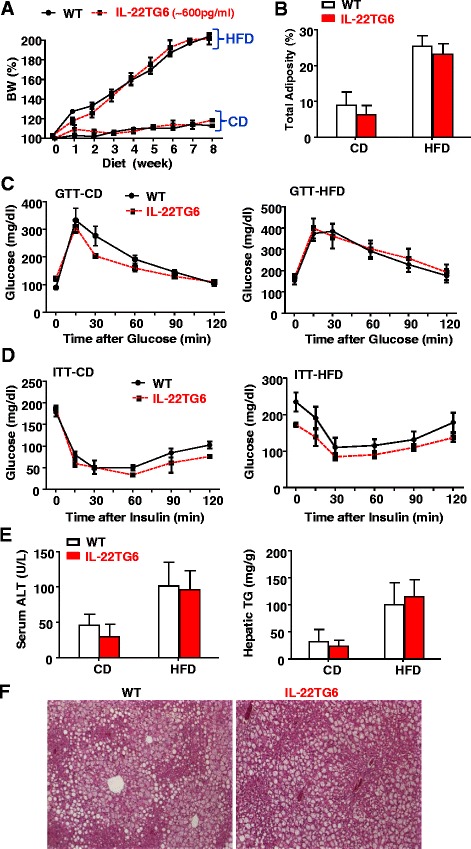
IL-22TG6 mice with high levels of circulating IL-22 (~600 pg/ml) develop the same degree of HFD-induced obesity, insulin resistance, and fatty liver as wild-type littermates. Mice were fed a CD or HFD for 8 weeks. **a** Body weights were measured. **b** Total adiposity. **c** Glucose tolerance test (GTT). **d** Insulin resistance test (ITT). **e** Serum ALT and hepatic TG levels were measured. **f** Representative H&E staining of liver tissues from 8-week HFD fed mice. Values represent the mean ± SEM (n = 6-14)

We have previously found that the IL-22TG8, TG9, and TG15 mice had much lower lean body weight [14], suggesting that super high IL-22 levels may cause cachexia. The IL-22TG6 with relatively high levels of circulating IL-22 had the same lean body weight as the wild-type littermate controls (Fig. 3a). Therefore, in the current study, HFD-induced obesity and its metabolic consequences were only examined in IL-22TG6 mice. After HFD feeding, IL-22TG6 mice gained comparable body weight and had the same total adiposity as WT littermate controls (Fig. 3a-b). Glucose tolerance test (GTT) and insulin tolerance test (ITT) analyses revealed that there were no differences in glucose tolerance or insulin sensitivity between CD- or HFD-fed WT and IL-22TG6 mice (Fig. 3c-d). Moreover, serum ALT levels, hepatic TG levels, and hepatic steatosis were also comparable in HFD-fed WT and IL-22TG6 mice (Fig. 3e-f).

### Chronic treatment with recombinant mouse IL-22 (rmIL-22) protein does not ameliorate HFD-induced obesity and the associated metabolic syndrome

In an early study, chronic treatment with rmIL-22 (300 ng/g, daily injection) (Generon Corporation, Shanghai, China) for 36 days ameliorated fatty liver but did not affect body weight, fasting glucose, and fasting insulin levels in HFD-fed mice [21], while a recent study reported that chronic treatment with low doses of rmIL-22 (R&D systems) (20 ng/g or 100 ng/g body weight, twice a week) ameliorated obesity and metabolic syndrome in HFD-fed mice [20]. One of the reasons for this discrepancy might be the different sources of rmIL-22 were used.

**Fig. 4:**
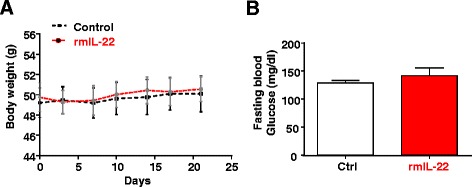
Chronic administration of rmIL-22 protein does not affect body weight and insulin resistance in HFD-fed mice. Mice were fed a HFD for 5 months and then injected with rmIL-22 (20 ng/g body weight, i.p. injection, twice a week) or saline for an additional four weeks. **a** Body weights were measured. **b** Fasting blood glucose levels. Values represent the mean ± SEM (n = 8)

To further clarify this discrepancy, we treated HFD-fed mice with rmIL-22 (R&D system) (20 ng/g body weight, twice a week) for 4 weeks. Our results revealed that such treatment did not affect body weight and fasting glucose levels (Fig. 4a-b) and insulin and glucose intolerance (data not shown).

### Administration of a single dose of the rmIL-22 protein reduces blood glucose levels in HFD-fed and streptozocin (STZ)-treated mice without affecting blood insulin levels: IL-22 activates STAT3 in acinar cells but not in islets in the pancreas

**Fig. 5:**
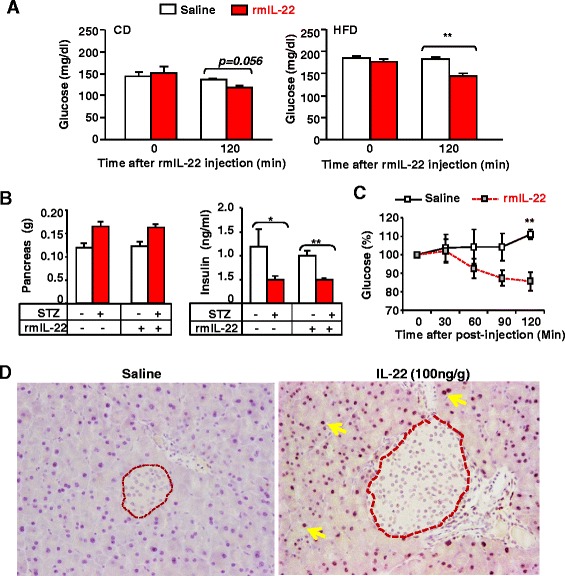
Injection of a single dose of recombinant mouse IL-22 (rmIL-22) protein reduces blood glucose levels in HFD- and streptozotocin (STZ)-treated mice. **a** Mice were fed a HFD for 8 weeks and then injected with saline or rmIL-22 for 2 h. Blood glucose levels were detected 120 min post IL-22 injection. **b**, **c** C57BL/6 mice were injected with STZ for 5 consecutive days. Twenty eight days later, mice were injected with saline or rmIL-22, and sacrificed 2 h later. Pancreas weights and insulin levels were measured (panel **b**). Glucose levels were measured at various time points post rmIL-22 injection (panel **c**). Values represent the mean ± SEM (n = 10). *P < 0.05 and **P < 0.01 compared with the corresponding saline treated groups. **d** C57BL/6 mice were treated with rmIL-22 for 2 h, pancreas tissues were collected for immunostaining with anti-pSTAT3 antibody. Representative positive pSTAT3 nuclei in acinar cells are indicated by yellow arrows but not in islets (indicated by dotted lines)

To explore whether pharmacologic doses of rmIL-22 have any acute beneficial metabolic effects, we treated mice with a single dose of the rmIL-22 protein. Administration of a single dose of rmIL-22 (1000 ng/g body weight) did not significantly affect body weight in mice fed a HFD or CD (data not shown). However, injection of a single dose of rmIL-22 significantly decreased fasting blood glucose levels in mice fed a HFD and decreased plasma glucose levels in mice fed a CD to a lesser extent (Fig. 5a).

Next, we tested whether IL-22 also reduces blood glucose levels in a model of type I diabetes induced via STZ injection. Injection of STZ caused pancreatic islet damage (data not shown) and decreased serum insulin levels in WT mice (Fig. 5b). Similar pancreatic islet damage was observed in STZ mice treated with or without IL-22 treatment (data not shown). Moreover, as illustrated in Fig. 5b-c, injection of a single dose of rmIL-22 did not affect the pancreas weight nor serum insulin levels in both vehicle- or STZ-treated groups but markedly reduced blood glucose levels in STZ-treated mice.

We and others previously reported that IL-22 treatment protects against cerulean-induced pancreatitis in mice by targeting pancreatic acinar cells [30, 31]. Interestingly, a recent study reported that IL-22 can directly target mouse and human pancreatic islet beta cells [20]. However, immunohistochemistry analyses revealed strong pSTAT3 staining in acinar cells but not in islets in the pancreas from IL-22-treated mice (Fig. 5d).

### IL-22 inhibits hepatic gluconeogenesis without affecting glucose uptake

**Fig. 6:**
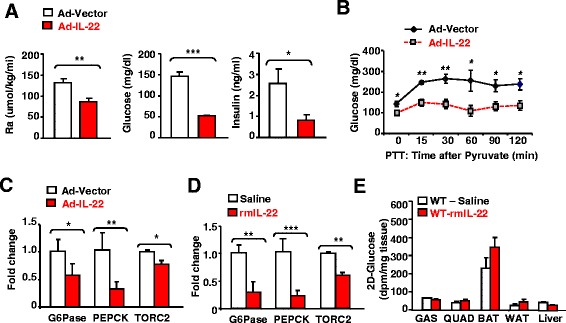
IL-22 inhibits hepatic gluconeogenesis without affecting glucose uptake *in vivo*. **a**-**c** Mice were fed a HFD for 8 weeks and then injected with ad-vector or ad-IL-22 for 5 days. A glucose tracer assay *in vivo* was performed. Glucose turnover rates and plasma glucose levels are shown (panel **a**). A pyruvate tolerance test (PTT) was performed (panel **b**). Real-time PCR analyses of gluconeogenic genes (panel **c**). **d**-**e** C57BL/6 mice were fed a HFD for 8 weeks and then fasted for 4 h, followed by treatment with saline or rmIL-22 (1 μg/g) for 2 h. Real-time PCR analyses of gluconeogenic genes (panel **d**). Two-deoxyglucose uptake experiments *in vivo* were performed (panel **e**). Values represent the mean ± SEM (n=6-10). *P < 0.05, **P < 0.01, and ***P < 0.001 compared with the corresponding ad-IL-22-treated or rmIL-22-treated groups

To explore the mechanisms by which IL-22 reduces blood glucose levels in HFD-fed mice, we performed an *in vivo* glucose turn-over assay and pyruvate tolerance test (PTT), an assay to determine hepatic gluconeogenesis *in vivo*. HFD-fed mice were injected with IL-22 adenovirus or control adenovirus prior to the glucose turn-over assay. Injection of Ad-IL-22 resulted in significant elevation of circulating IL-22 (~5000 pg/ml), and this elevation lasted for more than two weeks [10]. As illustrated in Fig. 6a, the glucose turn-over rate and blood glucose levels were markedly lower in ad-IL-22-treated mice compared with ad-vector-treated mice. Fig. 6b shows that blood glucose levels were significantly elevated after injection of pyruvate in ad-vector-treated mice; however, such elevation was not observed in ad-IL-22 injected mice, suggesting that injection of ad-IL-22 blocks hepatic gluconeogenesis. Indeed, expression of gluconeogenic genes, including G6Pase, PEPCK, and TORC2 (also known as CRTC2), was markedly reduced in ad-IL-22-treated mice compared with ad-vector-treated mice (Fig. 6c). In addition, administration of a single dose of rmIL-22 markedly suppressed hepatic expression of gluconeogenic genes (Fig. 6d).

Glucose uptake experiments showed that IL-22 treatment did not affect glucose uptake in the liver, muscle, and WAT tissues (Fig. 6e). There was a trend towards an increase in BAT in mice treated with rmIL-22, but it did not reach statistical significance.

### STAT3 and AMPK but not PI3/AKT contribute to IL-22 inhibition of hepatic gluconeogenesis in vitro

**Fig. 7:**
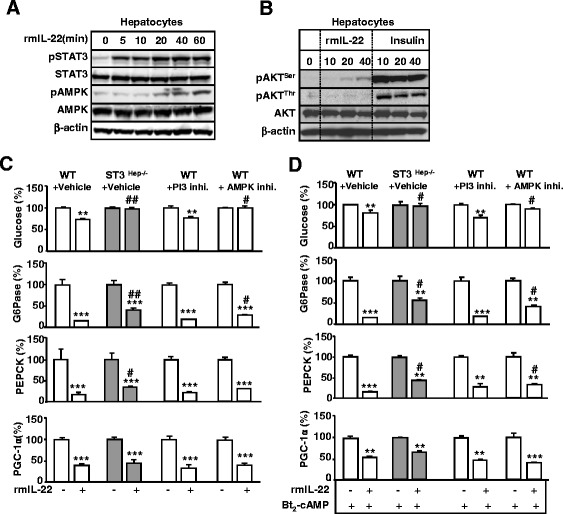
Treatment with rmIL-22 protein inhibits gluconeogenesis in primary mouse hepatocytes via STAT3- and AMPK-dependent mechanisms. **a** Western blot analyses of IL-22-treated primary mouse hepatocytes. **b** Western blot analyses of IL-22- or insulin-treated hepatocytes. **c** Primary wild-type mouse hepatocytes with pre-treated PI3K or AMPK inhibitors, followed by IL-22 treatment. Primary STAT3KO mouse hepatocytes were also treated with IL-22. **d** The same experiments as those in panel C except all cells were pre-treated with Bt2-cAMP. In panels **c** and **d**, glucose production and gene expression were analyzed and normalized to 100 % in hepatocytes without IL-22 treatment in each group. Values represent the mean ± SEM (n = 4). *P < 0.05, **P < 0.01, and ***P < 0.001 compared with the corresponding hepatocytes without rmIL-22 treatment. ^#^ *P* < 0.05 and ^##^ *P* < 0.01 compared with the corresponding hepatocytes from vehicle + WT mice with rmIL-22 treatment

To further understand the mechanisms underlying IL-22-mediated inhibition of hepatic gluconeogenesis, we examined the effects of IL-22 signaling pathways on hepatic gluconeogenesis and gluconeogenic genes in primary mouse hepatocytes. As shown in Figs. 7a-b, treatment with rmIL-22 predominantly induced the phosphorylation and activation of STAT3 and to a lesser extent induced pAKT and pAMPK activation in primary mouse hepatocytes. Compared to insulin stimulation, rmIL-22 induced much weaker pAKT activation in primary mouse hepatocytes. In addition, rmIL-22 treatment did not further enhance insulin activation of pAKT (data not shown).

Next, we investigated whether these signaling pathways contributed to the IL-22-mediated inhibition of glucose production in primary hepatocytes. As shown in Fig. 7c, treatment with rmIL-22 decreased basal glucose production, and expression of gluconeogeneic genes, including PEPCK, G6Pase, and PGC-1α, in WT hepatocytes. IL-22 inhibition of glucose production was completely abolished, whereas IL-22 inhibition of these genes was partially diminished in STAT3 knockout hepatocytes. Interestingly, treatment with AMPK inhibitor but not with the PI3 kinase inhibitor LY294002 partially diminished the IL-22 inhibition of glucose production and G6Pase gene expression in WT hepatocytes.

Similarly, treatment with rmIL-22 also decreased Bt2-cAMP-induced glucose production in WT mouse hepatocytes but not in STAT3 knockout hepatocytes. Such inhibition was also partially diminished in AMPK inhibitor-treated hepatocytes but not in PI3 inhibitor-treated hepatocytes (Fig. 7d). Treatment with rmIL-22 inhibited the Bt2-cAMP induction of PEPCK, G6Pase, and PGC-1α expression in WT mice (Fig. 7d). rmIL-22 inhibition of PEPCK and G6Pase but not PGC-1α was partially diminished in STAT3 knockout hepatocytes and AMPK inhibitor-treated hepatocytes (Fig. 7d).

## Discussion

As mentioned in the introduction, the effects of IL-22 on obesity and metabolic syndrome are very controversial. Here, we provide evidence that endogenous IL-22 or biologically active, high circulating levels of IL-22 do not affect HFD-induced obesity and its metabolic consequences, although IL-22 is able to inhibit hepatic gluconeogenesis in hepatocytes.

### Endogenous IL-22 does not play a role in modulating HFD-induced metabolic syndrome

It has been reported that serum levels of IL-22 in mice are decreased by HFD feeding from approximately 20 pg/ml in lean controls to approximately 5 pg/ml [28]. In our study, serum levels of IL-22 were also relatively low (<20 pg/ml) in both CD and HFD-fed mice (Fig. 1a). In addition, we found that IL-22TG6 mice, which have high circulating levels of IL-22 (~600 pg/ml), develop the same degree of HFD-induced obesity and its metabolic consequences as wild-type littermates. Thus, the very low levels of endogenous IL-22 are unlikely to contribute to the pathogenesis of HFD-induced obesity and its metabolic sequelae. This notion is further supported by our finding that HFD feeding induced the same degree of body weight gain, obesity, and insulin resistance in WT littermates and IL-22KO mice. Recently, Wang et al. [19] also reported that HFD feeding induced comparable levels of obesity in IL-22KO mice and WT mice, whereas IL-22R1KO mice were more susceptible to HFD-induced obesity and insulin resistance. Because IL-22R1 can combine not only with IL-10R2 to act as a functional IL-22R complex but can also interact with IL-20R2 to form a receptor for IL-20 and IL-24 [32], it is likely that IL-22R1 ligands other than IL-22 may play a role in ameliorating HFD-induced metabolic syndrome.

### Biologically active, high levels of IL-22 do not modulate HFD-induced obesity and its metabolic consequences

Super high levels of circulating IL-22 (4000–7000 pg/ml) in IL-22TG8 mice or in mice treated with ad-IL-22 caused marked body weight loss in lean mice [14, 18], suggesting that super high levels of IL-22 induce cachexia. At present, the mechanisms underlying IL-22-mediated cachexia remain unclear. It has been well-documented that a wide variety of cytokines can induce cachexia after prolonged production via multiple mechanisms, and these cytokines include TNF-α, IL-6, leukemia inhibitory factor (LIF), ciliary neurotrophic factor (CNTF) and interferon-γ (IFN-γ) [33]. It is likely that super high levels of IL-22 promote cachexia by using mechanisms similar to those used by these cytokines such as induction of strong acute phase response and subsequent chronic inflammation [18, 33]. Although it was reported that IL-22 can indirectly induce inflammation in chronic liver disease [17], hepatic and serum levels of IL-6, TNF-α, IL-1β were not elevated in mice treated with high doses of Ad-IL-22 [18] or in IL-22TG8 mice with super high levels of circulating IL-22 (~6000 pg/ml) (Park et al. unpublished data). This suggests that the cachectic effect of super high levels of IL-22 is not due to upregulation of the cachectic factors such as IL-6 and TNF-α.

Recently, Wang et al. [19] reported that chronic treatment of mice with high doses of IL-22Fc protein (50–100 μg/mouse, twice a week) for 6 weeks markedly reduced the body weights of HFD-fed mice. IL-22Fc has a long half-life (t_1/2_ = 3.02 days) and administration of high doses of IL-22Fc may result in sustained high levels of IL-22 *in vivo*. Although Wang et al. reported that IL-22Fc treatment reduced fat pad size in HFD-fed mice, they did not analyze body composition, nor did they examine the effect of IL-22Fc treatment on body weight in lean mice, so we can’t rule out the possibility that the weight-reducing effect of IL-22Fc treatment in HFD-fed mice was due to cachexia caused by super high levels of IL-22.

In an early study, chronic treatment with rmIL-22 (300 ng/g, daily for 36 days) improved fatty liver but did not affect body weight and the associated metabolic syndrome in HFD-fed mice [21]. This is consistent with our findings that relatively high levels of IL-22 in IL-22TG6 mice do not affect obesity. Lack of the anti-obesity effects of high levels of circulating IL-22 (600 pg/ml) in IL-22TG6 mice was very unlikely due to developing hepatic IL-22 resistance because IL-22TG6 mice were completely resistant to Con A-induced liver injury. Surprisingly, a recent study reported that chronic treatment with low doses of rmIL-22 markedly ameliorated obesity and metabolic syndrome in HFD-fed mice [20]; however, these results could not be reproduced in the present study.

### IL-22 regulates lipid and glucose metabolism

Previous studies have revealed that IL-22 ameliorates fatty liver disease by downregulating hepatic expression of several lipogenic genes [10, 21]. In the current study, we have demonstrated that IL-22 also regulates glucose metabolism via the inhibition of hepatic gluconeogenesis. First, injection of adeno-IL-22 inhibited glucose turn-over rate and gluconeogenesis in the liver. Second, treatment with rmIL-22 inhibited glucose production in primary mouse hepatocytes. Finally, treatment with IL-22 markedly reduced the expression of gluconeogenic genes, including PEPCK and G6Pase, in the liver *in vivo* and in hepatocytes *in vitro.* The STAT3, which is the major downstream of IL-22 signaling pathway, has been shown to inhibit hepatic lipogenesis and gluconeogenesis [34], which is in line with the effect of IL-22 on lipid and glucose metabolism in hepatocytes. In addition to the predominant activation of STAT3 in hepatocytes, IL-22 also activates, to a lesser extent, many other signaling pathways including AKT (as shown in Fig. 7 in this paper, and references [1–3]). Both STAT3 and AKT are known to inhibit hepatic gluconeogenesis [34, 35], but our results suggest that activation of STAT3 and not that of AKT is involved in the IL-22 suppression of hepatic gluconeogenesis. *In vitro* treatment with rmIL-22 inhibited glucose production in primary WT mouse hepatocytes but not in STAT3 knockout hepatocytes. Interestingly, although the effects of IL-22 on glucose production were completely absent in STAT3 knockout hepatocytes, its effect on gluconeogenic gene expression was only partially reduced, suggesting the involvement of additional signaling pathways in this latter effects. Despite an important role for AKT in inhibiting hepatic gluconeogenesis [35], inhibition of PI3/AKT by the PI3 kinase inhibitor LY294002 did not alter the effects of IL-22 on glucose production and gluconeogenic gene expression. The lack of a role for AKT in IL-22 inhibition of gluconeogenesis may be related to the fact that IL-22 only induced very weak AKT activation in hepatocytes.

In this study, we have demonstrated for the first time that IL-22 treatment activates AMPK in hepatocytes. AMPK has been shown to play an important role in inhibiting hepatic gluconeogenesis [36]. Our findings that the AMPK inhibitor compound C abolished the IL-22-mediated inhibition of glucose production and gluconeogenic gene expression suggest that the activation of AMPK is also involved in the IL-22-mediated inhibition of gluconeogenesis in hepatocytes.

### IL-22 treatment does not protect against STZ-induced islet damage and insulin reduction in mice

It is well documented that IL-22R1 is primarily expressed in epithelial cells, such as acinar cells in the pancreas [3, 30, 31]. Interestingly, Hasanin et al. recently reported that pancreatic beta cells express IL-22R1, and IL-22 administration suppresses ER stress and inflammation and promotes insulin secretion in beta cells [20]. However, several lines of evidence argue against this notion. First, blood insulin levels were lower in IL-22TG8 mice than in WT mice under HFD or normal chow feeding (Park et al. unpublished data). Second, administration of STZ causes beta cell damage and reduces insulin production, which was not averted by administration of rmIL-22. Third, STAT3, which is a major downstream signaling molecule activated by IL-22, mediates the cytoprotective functions of IL-22 [3]. Injection of IL-22 resulted in strong pSTAT3 activation in acinar cells but not in beta cells. This activation in acinar cells contributes to the protective effects exerted by IL-22 on acinar cells and pancreatitis [30, 31]. Although we did not detect STAT3 activation in beta cells from IL-22-treated mice, we can’t rule out that IL-22 may stimulate the STAT3 pathway at low levels, which may still have a beneficial effect under stress conditions, or the unlikely possibility that IL-22 activates other signaling pathways in beta cells. Further studies are needed to clarify the functions of IL-22 in beta cells in the pancreas.

In summary, our findings suggest that biologically active, high levels of IL-22 do not affect obesity and its metabolic consequence. Super high levels of IL-22 may cause cachexia and subsequently body weight loss. IL-22 inhibits hepatic gluconeogenesis via the activation of STAT3 and AMPK without affecting insulin production.

## Materials and methods

### Materials

Recombinant murine IL-22 protein (rmIL-22) was purchased from R&D system (Minneapolis, MN). All of the antibodies used for Western blot analysis were purchased from Cell Signaling (Danvers, MA).

### Mice

Eight- to ten-week-old male C57BL/6 N mice were purchased from the National Cancer Institute. Liver-specific IL-22 transgenic mice line 6 (IL-22TG6) on a C57BL/6 N background were generated as described previously [29]. IL-22TG-6 mice had relatively high serum levels of IL-22 (~600 pg/ml). IL-22 knockout out mice on a C57BL/6 background were kindly provided by Dr. Wenjun Ouyang (Genentech, San Francisco, CA, USA). All animal study protocols were reviewed and approved by the Institutional Animal Care and Use Committee of the National Institute on Alcohol Abuse and Alcoholism, National Institutes of Health.

### Diet-induced obesity

Eight- to ten-week-old male mice were fed either a control diet (CD) or a high-fat diet (HFD) for 8 to 12 weeks or 5 months. The HFD contained 34.0 % fat (60 % of calories), 26.3 % carbohydrates (20 % of calories), and 26.2 % protein (20 % of calories) as well as fiber, vitamins, and minerals (D12492, Research Diets, New Brunswick, NJ, USA). The CD contained 4.3 % fat (10 % of calories) (D12450B, Research Diets). The mice had free access to food and water. After feeding for various time periods, mice were euthanized and the liver and adipose tissues (gonadal, retroperitoneal, and subcutaneous) were removed, weighed, and snap-frozen. The adiposity index was calculated as the combined adipose tissue weight/carcass body weight × 100 %.

### Streptozotocin (STZ)-induced type I diabetes

Mice received 5 daily consecutive injections of 50 mg/kg STZ (dissolved in citrate buffer, pH 4.5, i.p. injection) in a volume that did not exceed 50 μl. Blood glucose levels were assessed after the last injection and then assessed weakly for 4 weeks.

### Glucose tolerance test (GTT), pyruvate tolerance test (PTT), and insulin tolerance test (ITT)

For the GTT, mice were fasted overnight, and tail vein blood was collected to measure glucose levels. The mice were then injected with glucose (2 g/kg i.p.), followed by the collection of tail vein blood and measurement of blood glucose levels at various time points. Blood glucose concentrations were measured using a Glucometer Contour (Bayer HealthCare, Mishawaka, IN, USA). For the ITT, mice were fasted for 6 h, and tail vein blood was collected to measure basal blood glucose levels. Mice were then injected with insulin (0.75 U/kg i.p., Eli Lilly), and blood glucose levels were measured at various time points. For the PTT test, mice were fasted for 16 h and injected with pyruvate (2 g/kg i.p.), and blood glucose levels were measured at various time points.

### Endogenous glucose production measurement in vivo

Basal glucose production was measured in restrained, conscious mice maintained on HFD for 8 weeks. Four days before the experiment, mice were anesthetized with 100 mg/kg ketamine and 10 mg/kg xylazine. A catheter was inserted through a lateral incision on the right side of the neck into the superior vena cava via the right internal jugular vein. The catheter was then sutured into place according to the protocol of MacLeod and Shapiro [37]. Experiments were started 3 h after fasting. The basal rates of glucose turnover were measured by continuous infusion of [3-^3^H]glucose (2 μCi bolus, then 0.05 μCi/min) for 180 min. Infusions were performed using microdialysis pumps (CMA 402/Microdialysis, Acton, MA, USA). Blood samples (20 μl) were collected via a small nick in the tail vein at 120, 150, 160, 170 and 180 min for the determination of plasma glucose and plasma [^3^H] glucose concentrations. An additional 10 μl of blood were collected at 120 and 180 min to measure plasma insulin concentrations by RIA (Millipore, St. Charles, MO, USA). The concentrations of glucose in the plasma were analyzed via the glucose oxidase method (YSI 2700 Select, Yellow Springs Instruments, Yellow Springs, OH, USA). The determination of plasma [3-^3^H] glucose was performed as described previously [38]. The appearance rates for glucose were calculated as the ratio of the [3-^3^H] glucose infusion rate (dpm/min) to the specific activity of the plasma glucose (dpm/μmol). Data are presented as average values during the last 30 min of the experiment.

### Glucose production in primary hepatocytes

Glucose production was determined by modified the protocol described by Foretz M et al. [39]. Briefly, primary mouse hepatocytes were isolated and plated in a 6-well collagen I coated plate (Biocoat plate, BD Biosciences, Bedford, MA) in DMEM containing antibiotics and 10 % FBS for 4 h, then switched to serum free DMEM with 100 nM dexamethasone (Dex) for 16 h prior to the measurement of glucose production and washed once with PBS. Cells were then incubated in glucose-free DMEM containing 10 mM lactate and 1 mM pyruvate with 100 uM Bt_2_-cAMP (Sigma Aldrich, St. Louis, MO), with or without IL-22, AKT-inhibitor, or compound C (EMD Chemicals Inc. Gibbstown, NJ). Glucose production was detected at 8 h incubation and measured by using 2300 STAT Plus Glucose Analyzer (YSI Life Sciences, Yellow Springs, OH). The value was normalized to the protein concentration.

### RNA extraction and real time quantitative RT-PCR

RNA was extracted from the liver tissue with Trizol (Invitrogen, Calsbad, CA) or QIAGEN RNAeasy kit (QIAGEN, Valencia, CA) according to the manufacturer’s instructions. mRNA expression of gluconeogenic genes was determined by real-time quantitative PCR, using a model 7500 PCR system (Applied Biosystems, Foster City, CA). Primers used in real-time PCR were described previously [39].

### Western blot analysis

Liver tissue was homogenized in the RIPA Lysis buffer containing proteinase cocktail, PMSF, and sodium orthovanadate (Santa Cruz Biotechnology, Inc. Santa Cruz, CA) and grinded by Precellys 24 (Bertin Technologies, France). To isolate protein extracts from primary hepatocytes, cells were washed twice with iced cold PBS, mixed with RIPA Lysis buffer, and then sonicated on ice for 20 s prior to collecting protein solution. Western blot analyses were performed and protein bands were visualized by enhanced chemiluminescence reaction (Amersham Pharmacia Biotech, Piscataway, NJ).

### Blood chemistry

Serum ALT levels were measured using chemistry analyzer (IDEXX Catalyst Dx, IDEXX Laboratories, Westbrook, ME). Serum insulin levels were determined using an ELISA kit (ALPCO Diagnostics, Salem, NH). Serum IL-22 levels were measured by an ELISA kit (R&D system).

### Hepatic triglyceride contents

Chloroform/methanol (2:1) solution was used for lipid extraction from the total liver. Extracted lipid was then dissolved in 5 % triton X-100 solution and hepatic triglyceride levels was measured using EnzyChrom™ triglyceride assay kit (BioAssay Systems, Hayward, CA).

### Histopathology

For general histological analysis, liver tissues were fixed in the 10 % neutralized formalin solution and embedded in the paraffin. Tissues were cut 4-um thickness and stained with hematoxylin and eosin (H&E). For Oil red O staining for fat accumulation, frozen liver tissues were cut for 10 μm sections with cryostat and stained with pre-warmed Oil Red O solution (Vector Laboratories, Burlingame, CA) for 10 min, rinsed in water, and then counterstained with Mayer’s hematoxylin, and analyzed by light microscopy.

### Administration of mice with IL-22 adenovirus

IL-22 adenovirus was made by cloning mouse IL-22 cDNA (544 bp) into the pENTR/D-TOPO system (Invitrogen), followed by using Invitrogen Gateway system to perform a LR reaction with pAd/CMV/V5-DEST to make the expression vector pAd/CMV/mIL-22. Mice were injected (intravenously) with adenovirus-IL-22 (2 × 10^8^ pfu) or adenovirus-empty vector (2 × 10^8^ pfu).

### Statistical analysis

Data are expressed as the mean ± SEM. To compare values obtained from two groups, Student’s *t*-test was performed. To compare values obtained from three or more groups, one-way ANOVA was performed followed by Tukey’s post-hoc test. A value of *P* < 0.05 was considered significant.

## Abbreviations

Adeno-IL-22: IL-22 adenovirus
Adeno-vector: Control vector adenovirus
AMPK: Adenosine monophosphate-activated protein kinase
G6Pase: Glucose-6-phospatase
GTT: Glucose tolerance test
IL-22: Interleukin-22
ITT: Insulin tolerance test
PEPCK: Phosphoenolpyruvate carboxykinase
PGC-1α: Peroxisome proliferator activated receptor gamma coactivator 1-alpha
PI3 Kinase: Phospoinositide 3-kinase
PTT: Pyruvate tolerance test
TG: Transgenic
STAT3: signal transducer and activator of transcription 3
STAT3^Hep−/−^ mice: Hepatocyte-specific STAT3 knockout mice
